# Magnetic resonance imaging of white matter response to diesel exhaust particles

**DOI:** 10.21203/rs.3.rs-3087503/v1

**Published:** 2023-07-18

**Authors:** Ararat Chakhoyan, Kristina Shkrkova, Saman Sizdahkhani, Mikko T. Huuskonen, Krista Lamorie-Foote, Arnold Diaz, Selena Chen, Qinghai Liu, Carla D’Agostino, Hongqiao Zhang, Wendy J. Mack, Constantinos Sioutas, Caleb E. Finch, Berislav Zlokovic, William J. Mack

**Affiliations:** Zilkha Neurogenetic Institute, Keck School of Medicine, University of Southern California; Zilkha Neurogenetic Institute, Keck School of Medicine, University of Southern California; Zilkha Neurogenetic Institute, Keck School of Medicine, University of Southern California; Zilkha Neurogenetic Institute, Keck School of Medicine, University of Southern California; Zilkha Neurogenetic Institute, Keck School of Medicine, University of Southern California; Leonard Davis School of Gerontology, University of Southern California; Zilkha Neurogenetic Institute, Keck School of Medicine, University of Southern California; Zilkha Neurogenetic Institute, Keck School of Medicine, University of Southern California; Leonard Davis School of Gerontology, University of Southern California; Leonard Davis School of Gerontology, University of Southern California; Department of Population and Public Health Sciences, Keck School of Medicine; Viterbi School of Engineering, University of Southern California; Leonard Davis School of Gerontology, University of Southern California; Zilkha Neurogenetic Institute, Keck School of Medicine, University of Southern California; Zilkha Neurogenetic Institute, Keck School of Medicine, University of Southern California

## Abstract

Air pollution is associated with risks of dementia and accelerated cognitive decline. Rodent air pollution models have shown white matter vulnerability. This study uses diffusion tensor imaging (DTI) to quantify changes to white matter microstructure and tractography in multiple myelinated regions after exposure to diesel exhaust particulate (DEP). Adult C57BL/6 male mice were exposed to re-aerosolized DEP (NIST SRM 2975) at a concentration of 100 ug/m^3^ for 200 hours. Ex-vivo MRI analysis and fractional anisotropy (FA)-aided white matter tractography were conducted to study the effect of DEP exposure on the brain white matter tracts. Immunohistochemistry was used to assess myelin and axonal structure. DEP exposure for 8 weeks altered myelin composition in multiple regions. Diffusion tensor imaging (DTI) showed decreased FA in the corpus callosum (30%), external capsule (15%), internal capsule (15%), and cingulum (31 %). Separate immunohistochemistry analyses confirmed prior findings. Myelin basic protein (MBP) was decreased (corpus callosum: 28%, external capsule: 29%), and degraded MPB increased (corpus callosum: 32%, external capsule: 53%) in the DEP group. White matter is highly susceptible to chronic DEP exposure. This study demonstrates the utility of DTI as a neuroanatomical tool in the context of air pollution and white matter myelin vulnerability.

## Introduction

Clinical and experimental studies suggest that brain white matter may be particularly susceptible to the neurotoxic effects of air pollution during early development and later life.^1–3^ An analysis of the Generation R study determined that higher levels of PM_2.5_ exposure from birth to age five were associated with altered white matter tract microstructure (lower fractional anisotropy and higher mean diffusivity) in preadolescents.^4^ In a murine model, early postnatal exposure to ultrafine particulate matter correlated with white matter hypomyelination and reduced corpus callosum size.^5^ A prospective MRI analysis of older women without dementia from the Women’s Health Initiative Memory Study (WHIMS) demonstrated an association between greater PM_2.5_ exposure and decreased white matter volume in the frontal, parietal, and temporal association regions and in the corpus callosum.^3^ There were no significant associations between PM_2.5_ exposure and gray matter or hippocampal volumes. Our murine models (C57BL/6) have shown histological white matter injury in the corpus callosum and internal capsule following ultrafine particulate matter exposure.^6,7^ However, in-vivo MR imaging did not demonstrate nPM-induced changes in corpus callosum size, fractional anisotropy (FA), or apparent diffusion coefficient (ADC).^6,8^ More sensitive MR measurements may reveal early changes in white matter connectivity. Diffusion tensor images (DTI) can show loss of fractional anisotropy in normal-appearing white matter. This study uses *ex-vivo* DTI to examine white matter fractional anisotropy at high spatial resolution and signal-to-noise ratio to determine how DEP exposure may impact multiple white matter regions. Histochemical studies are used to determine myelin and axonal integrity.

## Methods

### Animal procedures

All experiments were reviewed and performed under protocols approved by the Institutional Animal Care and Use Committee at the University of Southern California in accordance with the Guide for the Care and Use of Laboratory Animals (NIH). The study is reported in accordance with ARRIVE guidelines. C57BL/6 male and female mice (Jackson Laboratories, USA) aged 10 weeks, were assigned randomly to filtered Air or DEP exposure groups. All animals were housed on a 12-h light (6 am to 6 pm)/12-h dark cycle with access to food and water *ad libitum*. No adverse effects on animals’ well-being were observed using commercial diesel exhaust particulate matter. Throughout the study we monitored loss of appetite, dramatic changes to body weight, and behavioral changes in grooming and activity. Within 48 hours following the exposure, mice were put under deep anesthesia with isoflurane and humanely euthanized by cardiectomy with saline perfusion. Brain tissue was carefully isolated, dissected, and stored for *ex-vivo* MRI and immunofluorescence. All sample processing was performed in random order. All animals were included in the analysis. A total of 61 mice (27 Filter Air and 34 DEP) were analyzed for immunohistochemistry. Samples were blinded for analysis and two observers were averaged. A total of 14 mice were used for *ex-vivo* MRI (7 Filter Air and 7 DEP).

### Exposure design to diesel exhaust particles

Standardized DEP (NIST SRM 2975) was obtained from the National Institute of Science and Technology. Particles were suspended in ultrapure Milli-Q water (at an aqueous concentration of 200 ug/ml) by 30 min sonication and re-aerosolized at a concentration of 100 pg/m^3^. During animal exposure, prepared DEP suspension was re-aerosolized into the exposure chambers by means of a HOPE jet nebulizer (Model 1131; B&B Medical Technologies, Carlsbad, CA). The aerosolized stream was mixed with clean air filtered by HEPA (1214; Pall Laboratory, Port Washington, NY). The re-aerosolized DEP was then drawn through a silica gel diffusion dryer (Model 3620, TSI Inc., USA) to remove excess water followed by a Po-210 neutralizer to neutralize electrical charge (Model 2U500, NRD Inc., USA). Particles then entered the exposure chamber at a flow rate of 2.5 Ipm. We continuously tracked and measured (5-minute intervals) variations in particles size and mass by TSI DustTrak (Model 8520, TSI Inc., USA). After sonication, DEP suspensions had a mean size of 57 nm, and after re-aerosolization, 69% particles were < 100 nm while the mean diameter was 65 nm. The duration of diesel exhaust exposure in this study was 8 weeks (200 hours total). Animals were exposed to filtered air or DEP for 5 hours a day, 3 times a week. The chamber size is 52 cm in length, 16 cm in height, and 32 cm in width, and each chamber has 9 separate meshed boxes that can house up to 27 mice (3 mice per box) from the same exposure group. Further details on DEP re-aerosolization system and characterization were previously reported.^9^

### Ex-vivo mouse brain DTI imaging

MRI was performed using a cryogen-free 7T MRI scanner (MR Solutions) at the Zilkha Neurological Institute (Department of Physiology and Neuroscience, Keck School of Medicine, University of Southern California, Los Angeles, CA). Mice were anesthetized with 100 mg/kg ketamine and 10 mg/kg xylazine i.p. Immediately after, trans-cardial perfusion was performed by 30 ml PBS (0.005 M EDTA), followed with 30 ml of 4% paraformaldehyde (PFA) in PBS. Brains were then immersed in 0.2mM Pro-Hence for 48 hours prior to enhance MRI signal during acquisition scheme. Spin-echo, echo-planner imaging (SE-EPI) sequence was used to acquire diffusion weighted imaging (DWI) with the following parameters: (TR/TE, 4,000/23ms; flip angle 90°, 1 average; 65 diffusion sampling directions; b-value,1800s/mm^2^; FOV, 0.12×0.12; slice thickness, 0.5mm and matrix size, 150×150). Thirty-two contiguous slices were acquired for a total acquisition time of 10h50min.

### Mathematical modeling of diffusivity tensor-derived parameters

From raw DWI datasets, mathematical modeling of diffusivity tensor-derived maps was computed using DSI Studio (http://dsi-studio.labsolver.org/). Our analysis was focused on fractional anisotropy (FA) measures as modeled below:

$$FA=\sqrt{\frac{3}{2}}\frac{\sqrt{{\left(\lambda 1-\text{MD}\right)}^{2}+{\left(\lambda 2-\text{MD}\right)}^{2}+{\left(\lambda 3-\text{MD}\right)}^{2}}}{\sqrt{\lambda 1^{2}+\lambda 2^{2}+}\lambda 3^{2}}$$

Where MD refers to mean diffusivity, and *λ*1, *λ*2 and *λ*3 represent the three main eigenvalues of the tensor.

### Template creation and image registration

For spatial registration and group comparison, a study-specific b_0_ template was created from DWI images (n = 14). We used an iterative group-wise normalization procedure (SyN) as implemented in buildtemplateparallel.sh script v0.0.14 (Advanced Normalization Tools, ANTs).^10^ Next, individual b_0_ images were registered to the template using a fully automated linear registration tool (FLIRT, fsl.fmrib.ox.ac.uk).^11^ The registration matrix was applied to each consecutive FA map. Finally, mean FA maps were computed for the filtered air and DEP groups.

### FA-aided white matter tractography

Corpus callosum was used as the seed ROI to generate FA-aided white matter tractography as implemented in a previous study.^8^ Automatic white matter tracking was generated using DSI studio with a generalized version of the deterministic tracking algorithm.^12^ After Eddy-current correction, motion correction was applied to eliminate scan-induced biases. Before running fiber reconstruction, the b-table was visually checked and flipped in either x, y, or z directions to obtain the best fiber coherence. A DTI reconstruction method was selected in our processing pipeline. The corpus callosum region was manually delineated and used as a seed region with a fractional anisotropy threshold of 0.3 (tracking index), maximum angular threshold of 60°, with minimum and maximum track lengths of 0 and 100mm, respectively. Directional color coding was selected for visual rendering with maximal 5000 visible tracts.

### Immunofluorescence

Tissues for immunofluorescent analysis were stored in 4% cold paraformaldehyde for 24 hours and subsequently transferred to 70% alcohol. Brain tissue (right hemisphere cut at bregma) was fixed in paraffin and sliced in the coronal plane at 5-micron thickness. Paraffin cut tissue sections were deparaffinized and rehydrated by immersing the slides in xylene, multiple alcohol concentrations (100 – 70%) and distilled water. The antigen retrieval step was performed by heating the tissue with sodium citrate. After antigen retrieval, sections were blocked in 5% donkey serum for 1 hour. Immunofluorescent staining was performed overnight with primary antibodies of degraded myelin basic protein (dMBP), myelin basic protein (MBP), and anti-neurofilament antibody (SMI-312). Secondary antibody was matched to primary antibody and stained for 1 hour at room temperature. Nuclei 4′,6-diamidino-2-phenylindole (DAPI) stain was performed for 10 minutes. Stained tissues were imaged using a fluorescent microscope (BZ-X800, Keyence, USA) at 40x magnification to visualize corpus collosum, cingulum, and external capsule. Total fluorescent density in the regions of interest (corpus callosum, cingulum, external capsule) were quantified using ImageJ. Images were blinded prior to being assessed by two independent observers. The internal capsule region was not available for immunofluorescent analysis due to its proximity to the edge of the embedded hemisphere and inability to control for irregularities in the tissue dissection.

### Behavior Forced Swim Test

We used the Forced Swim Test to evaluate the effect of DEP exposure on behavior of stress resistance and adaptability. On the testing day after exposure completion, mice were weighed and placed in a water filled cylinder one by one with water temperature maintained at 25°C. The water levels were kept consistent between animals. During the 6-minute exposure (2 min for acclimatization in the beginning) in the water tank, mice behavior was tracked. We quantified time spent immobile in the water tank as well as total distance swam. Mice were returned to home cages after the test.^13^

### Statistical analysis

Data are shown as mean ± standard deviation with statistical significance judged at 2-sided alpha of 0.05. The normality of distributions was checked with a Kolmogorov-Smirnov test. Independent t-tests compared means between the Filtered Air and DEP groups. Comparisons of measures over brain regions within study groups used repeated measures ANOVA with post-hoc pairwise comparisons, adjusted for multiple testing. All statistical analyses used SPSS (version 28) and GraphPad Prism (version 9).

## Results

### Imaging

**Figure 1:**
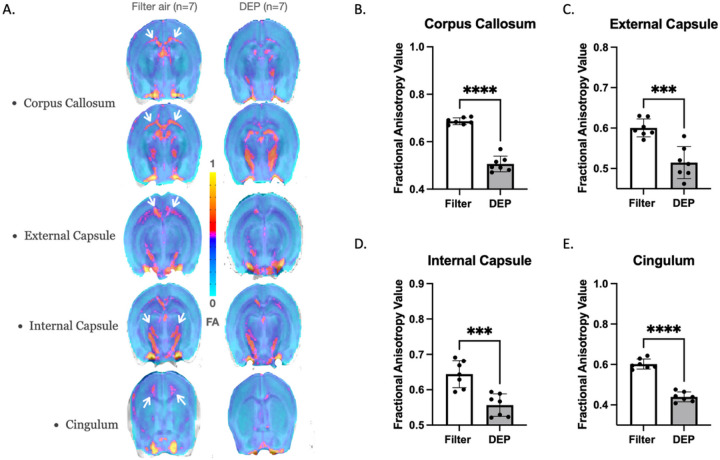
White Matter Fractional Anisotropy Mice were exposed to DEP at 100 ug/m^3^ for 8 weeks. White bars represent the filtered air group and grey bars represent the DEP group. A. FA group mapping within white matter regions: corpus callosum, external capsule, internal capsule, and cingulum. The small white arrows indicate regions of interests with highest FA reductions. DEP-exposed mice demonstrated significant FA decreases within: B. Corpus Callosum; C. External Capsule; D. Internal Capsule; D. Cingulum. *** p<0.001, **** p<0.0001 compared with filter air group of same exposure time. Error bars represent SD. N=7 in each group.

Using *ex-vivo* DTI, we demonstrated that DEP-exposed mice had white matter microstructural damage (when compared to filter air-exposed mice) in the corpus callosum, external capsule, internal capsule, and cingulum. Figure 1A illustrates mean FA group maps of 7 mice exposed to either filter air or DEP. Following DEP exposure, FA was reduced by 30% in the corpus callosum (0.51 ± 0.03 SD vs. 0.69 ± 0.01 SD, p < 0.0001), 15% in the external capsule (0.51 ± 0.04 vs. 0.6 ± 0.02, p = 0.0003), 15% in the internal capsule (0.56 ± 0.03 SD vs. 0.64 ± 0.04 SD, p = 0.0005), and 31 % in the cingulum (0.44 ± 0.02 SD vs. 0.60 ± 0.02 SD, p < 0.0001). Within the DEP group the internal capsule showed an asymmetric reduction of greater FA values in the left hemisphere compared to the right hemisphere. Mean FA did not differ between the DEP and filtered air groups in cortical gray matter (cortex) (0.29 ± 0.04 SD vs. 0.31 ± 0.05 SD, p > 0.05) or hippocampus (0.43 ± 0.08 SD vs. 0.41 ± 0.12 SD, p > 0.05).

**Figure 2:**
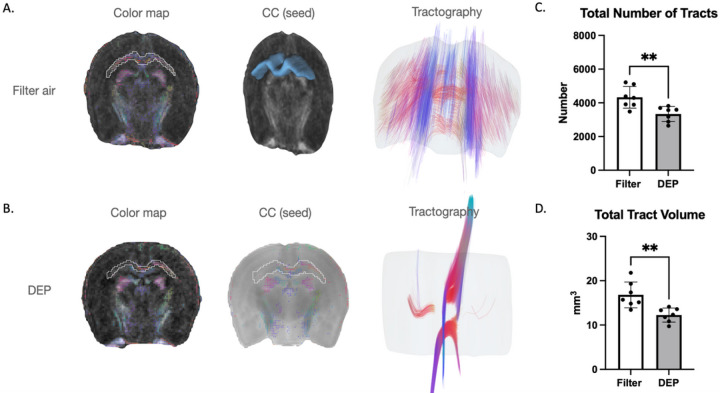
White Matter Tractography White bars represent the filtered air group and grey bars represent the DEP group. A. Interhemispheric connections and antero-posterior layers of corpus callosum in the filtered air group. B. Disconnection of interhemispheric white matter tracts in the DEP group. C. Total number of tracts. D. Total tract volume. ** p<0.01. Error bars represent SD. N=7 in each group.

Next, we examined FA-aided white matter tractography in the corpus callosum (Fig. 2). DEP exposure caused degradation of white matter connectivity (compared to filtered air). Tract volume was decreased by 30% (12.2 ± 1.6 SD vs. 16.8 ± 2.9 SD, p = 0.003) and total tract number decreased by 25% in the DEP group (3339.2 ± 446.2 SD vs. 4327.8 ± 644.3 SD, p = 0.006). The interhemispheric connections and antero-posterior layers of the corpus callosum are well structured tracts. DEP exposure was associated with substantially lower FA values that may represent disconnection of interhemispheric white matter tracts (Fig. 1). DEP exposure was associated with asymmetry in the corpus callosum; left hemisphere anterior and posterior layers were well preserved while right hemisphere layers demonstrated more damage/disorganization (Fig. 1).

### Immunofluorescence

**Figure 3:**
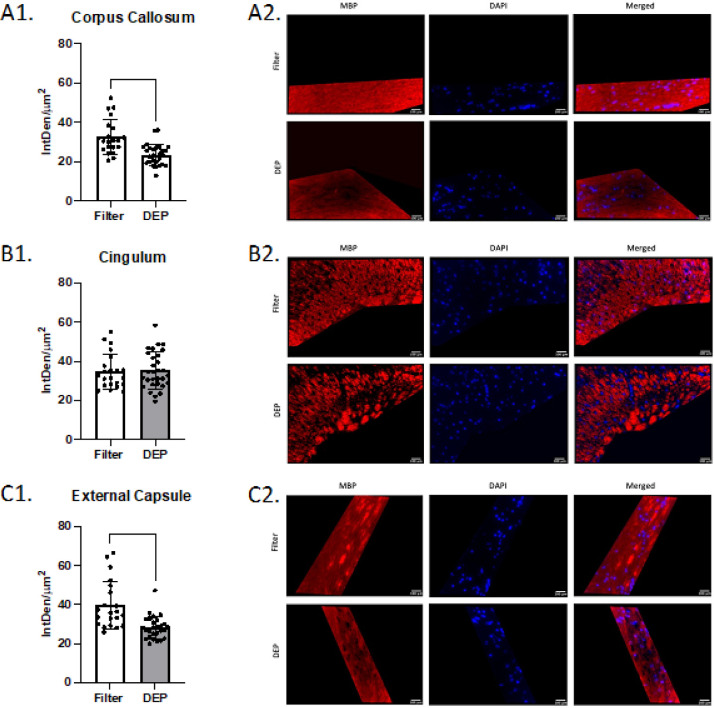
White Matter Myelin Basic Protein (MBP) Immunofluorescence White bars represent the filtered air group and grey bars represent the DEP group. A1. MBP total fluorescence in corpus callosum; A2. Representative corpus callosum images. B1. MBP total fluorescence in Cingulum; B2. Representative Cingulum images. C1. MBP total fluorescence in External Capsule; C2. Representative External Capsule images. **** p<0.0001 compared with filter air group of same exposure time. Error bars represent SD. N=11–19 per group.

**Figure 4:**
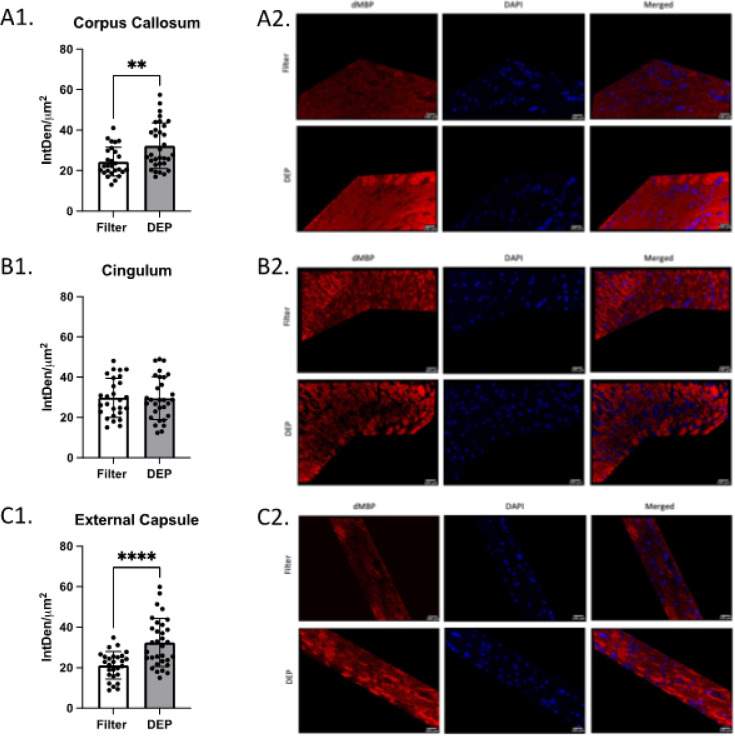
White Matter Degraded Myelin Basic Protein (dMBP) Immunofluorescence White bars represent the filtered air group and grey bars represent the DEP group. A1. dMBP total fluorescence in the Corpus Callosum; A2. Representative Corpus Callosum images. B1. dMBP total fluorescence in Cingulum; B2. Representative Cingulum images. C1. dMBP total fluorescence in External Capsule; C2. Representative External Capsule images. ** p<0.01, **** p<0.0001 compared with filter air group of same exposure time, Error bars represent SD. N=11–19 per group.

**Figure 5:**
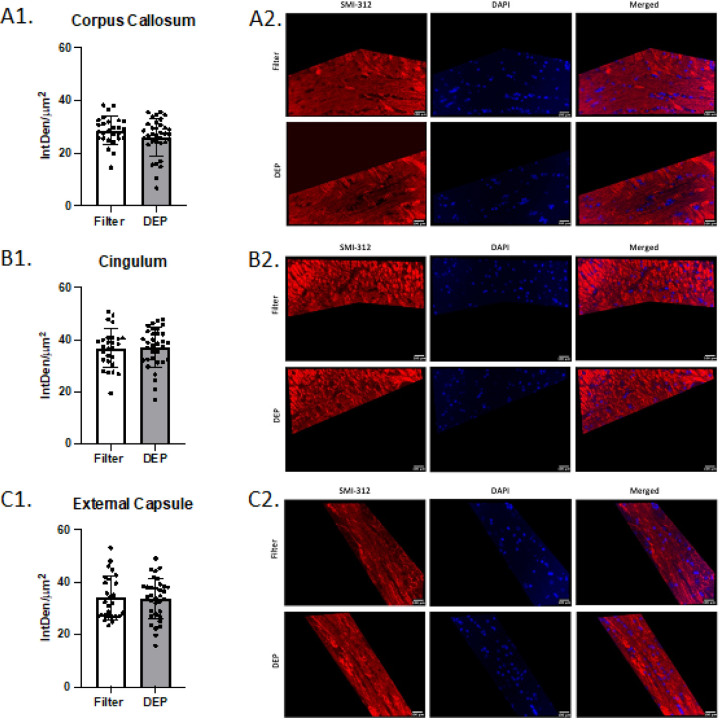
White Matter Neurofilament (SMI-312) Immunofluorescence White bars represent the filtered air group and grey bars represent the DEP group. A1. SMI-312 total fluorescence in Corpus Callosum; A2. Representative Corpus Callosum images. B1. SMI-312total fluorescence in Cingulum; B2. Representative Cingulum images. C1. SMI-312 total fluorescence in External Capsule; C2. Representative External Capsule images. Error bars represent SD. N=11–19 per group.

Mice exposed to DEP at 100 ug/m^3^ had significantly lower total fluorescent density (IntDen/um^2^) of MBP in the corpus callosum (32.6 ± 8.8 SD vs 23.6 ± 5.4 SD, p < 0.0001) and external capsule (39.7 ± 12.2 SD vs 28.3 ± 5.8 SD, p < 0.0001) than mice exposed to filtered air. No group differences were observed in the cingulum (p = 0.83, Fig. 3). Compared to the filtered air group, mice exposed to DEP had significantly higher total fluorescent density of dMBP in the corpus callosum (24.4 ± 7.2 SD vs 32.3 ± 11.2 SD, p = 0.003) and external capsule (21.2 ± 6.8 SD vs 32.4 ± 11.8 SD, p < 0.0001). No group difference was observed in the cingulum (p = 0.92, Fig. 4). No difference between filtered air and DEP was observed in corpus callosum, cingulum, and external capsule for SMI-312 (Fig. 5). No sex differences between males and females in total fluorescent density of dMBP MBP and SMI-312 markers were observed.

### Forced Swim Test

**Figure 6:**
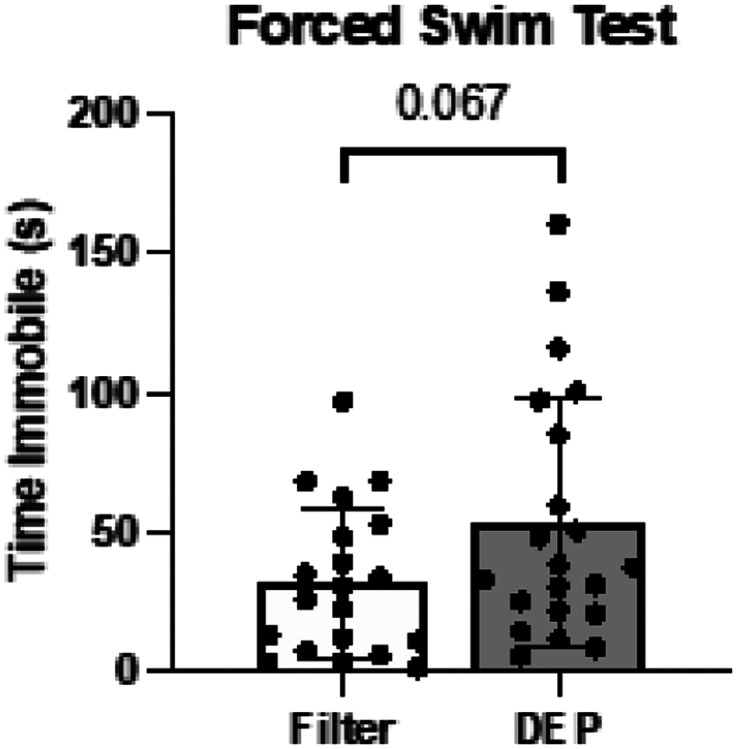
Forced Swim Test White bars represent the filtered air group and grey bars represent the DEP group. Mice underwent behavioral testing after exposure. Error bars represent SD. N=20–21 per group.

On the Forced Swim Test after the completion of Filter or DEP exposure mice showed only a trend towards higher immobility time in the water, a measure of reduced stress resistance and adaptability, in DEP group compared to Filter, without statistically significant difference observed (p = 0.067) (Fig. 6).

## Discussion

Human MR imaging studies have established associations between air pollution exposure, white matter injury, and decreases in functional brain connectivity.^1,3,14^ Structural brain MRI scans on a cohort of older women enrolled in the Women’s Health Initiative Memory Study demonstrated an association between greater PM_2.5_ levels and decreased white matter volumes.^3^ MR imaging on a cohort of children living in a heavily polluted metropolitan region of Mexico City showed increased prefrontal white matter hyperintensities and white matter volume loss in bilateral temporal and right parietal regions when compared to children from a less polluted area. These MR findings were accompanied by cognitive deficits in parietal and temporal function.^1^ A small, randomized crossover study suggested that short term (2h) diesel exhaust exposure resulted in decreases in connectivity in the default mode network on functional MR imaging. This localized to the right temporal and occipital lobes.^14^

Murine chronic PM_0.2_ exposure results in reproducible myelin degradation on histopathology.^6,7^ However, previous in-vivo MR imaging suggested no significant change in corpus callosum size or structure following 150h ultrafine PM exposure.^6,8^ With longer acquisition time, higher spatial resolution, and increased angular *ex-vivo* DTI analysis, the current study demonstrated decreased FA in the corpus callosum, external capsule, internal capsule, and cingulum. These changes correlated with FA-aided white matter tractography and revealed pronounced degradation of white matter connectivity (tract number and volume) in the DEP group. Anterior, medial, and posterior white matter regions were all susceptible to DEP-induced injury. Aberrant corpus callosum fibers did not cross the midplane, resulting in inter-hemispheric corpus callosum bundle disconnection. Irregular fibers within the longitudinal bundles of Probst appeared more affected in the right hemisphere. The ex-vivo white matter changes correlated with histochemical evidence (dMBP, MBP) of injury from this and prior studies.^2,6,7^ Indeed, the relationship between fractional anisotropy (FA) and histological assessments of myelin indicates that certain alterations in FA may be attributed to heightened levels of myelination.^15 16^

*Ex*-vivo diffusion tensor imaging demonstrated microstructural changes in white matter tracts and myelin sheaths that were not apparent on previous *in*-vivo MR imaging studies. DTI measures the magnitude of water diffusion in tissue structures with directional dependence. The presence of myelin has been shown to cause anisotropic diffusion in the white matter, with more rapid diffusion along the axis of myelinated axons than perpendicular to the axons. This directional dependence has been used to quantify the degree of myelination in the white matter and map the distribution of myelinated axons by FA.^17^ DTI techniques have been validated for the assessment of white matter orientation and tractography.^18,19^ In a previous study, 3D whole-brain immunolabeling of MBP and DTI measures demonstrated a strong sensitivity of the FA metric to myelinated white matter structures including the corpus collosum and anterior commissure.^20^

Our previous murine studies suggest that PM-induced white matter injury occurs prior to neuronal loss. One hundred fifty hours of nPM exposure resulted in decreased oligodendrocyte count in the corpus callosum, but not neuronal cell count in the frontal cortex.^21^ White matter microglia are more rapidly activated following DEP exposure than those in the adjacent gray matter. Following a single 5-hour DEP exposure, microglial activation (IBA-1 intensity) was increased in the corpus callosum, whereas IBA-1 cell count and soma area were not increased in the frontal cortex. Following 200h DEP exposure, Iba1-positive cell count showed the greatest increase in cortical layer 4 and microglial soma area the greatest increase in cortical layers 2–5. These cortical layers have important inputs and outputs through white matter tracts. The corpus callosum receives most axonal projections from layers 2, 3 and 5, while cortical layer 4 receives sensory inputs from subcortical structures and white matter regions.^22^

We confirmed by histochemistry that elevated levels of degraded myelin basic protein (dMBP) were present in the corpus callosum^23^ and demonstrated a similar increase in the external capsule following 8-week DEP exposure. We also showed reduction of MBP (healthy myelin) in both the corpus collosum and the internal capsule. The immunofluorescent findings in the cingulum, however, did not parallel our ex-vivo MRI data; the dMBP and MBP stains did not demonstrate myelin injury in the DEP group. This may be due to decreased stain sensitivity secondary to grey matter density in the cingulum.^24^ We also did not observe a DEP exposure effect on neurofilament peptides (SMI-312 stain) in the corpus callosum, cingulum, or external capsule. This negative finding is consistent with our previous nPM exposure studies.^8^ This indicates that sub-chronic or chronic air pollution exposure may not directly impact axonal architecture, but rather may inhibit white matter function through effects on myelin integrity. The lack of overt group differences on forced swim test supports the subtle nature of the white matter changes. However, the DEP exposed mice do tend to have longer immobility times. Our previous studies have shown that mice exhibit deficits on Novel Object in Context (NOIC) testing following 150h nPM exposure.^25^ While spatial reference memory is typically thought to be reliant on hippocampal function, injury to para-hippocampal regions, connecting fibers, and communicating white matter tracts can contribute.^26^ Our prior studies demonstrate PM-induced myelin injury in the hippocampal 01 region^27^ in addition to the corpus callosum and internal capsule.^6,7^

This study has limitations. *Ex-vivo* DTI is performed in fixed brains, in which tissue temperature is lower than in *in-vivo* experiments. This translates to decreased diffusivities of scalar indices, especially mean, axial, and radial diffusivities.^28^ Previous studies applied temperature corrections; however, diffusivity differences persisted^29^ while fractional anisotropy, tract length, and fiber densities remained unaltered *ex-vivo*.^30,31^ In this study, we investigated the total number of white matter tracts and tract volume. Global connectivity level and inter-hemispheric/intra-hemispheric structural connections were not specifically investigated. Given the complex relationship between DTI-derived FA maps and myelin, many variables including the length and thickness of the myelin sheath may contribute to FA measurements. Additionally, tissue fixation protocols have been reported to alter diffusion properties in the CNS.^32^ The reason behind the laterality of DTI tract damage (right greater than left) is unclear. This may have resulted from a relatively small sample size. However, it is notable that the children exposed to high levels of pollution in Mexico City exhibited white matter loss in the bilateral temporal and right parietal regions.^1^ Further, in the functional brain connectivity study, the Filtered Air group demonstrated greater DMN functional connectivity localized in the right middle temporal gyrus and occipital fusiform gyrus post exposure, whereas the DEP exposure group did not.^14^

This study demonstrates the neurotoxic effects of chronic DEP exposure on white matter tracts, especially the highly vulnerable myelin sheath. These changes parallel the early myelin injury evident in histochemical studies and do not affect axonal architecture. The ability of *ex*-vivo DTI imaging to detect white matter microstructural changes has the potential to enhance our understanding of air pollution effects on the brain in animal and human studies. Further experiments with greater sample size, expanded imaging parameters, and the use of mouse strains with neurodegenerative phenotypes will help delineate the impact of air pollution on white matter injury and dementia.

## Data Availability

Data from all scientific experiments described in this publication will be pulled, labelled, and organized in a master data guide by laboratory technicians and graduate students within the laboratories of listed principal investigators. All data sharing requests can be addressed to William Mack, MD (William.Mack@med.usc.edu) and will be reviewed in a timely manner by the main authors.
